# Supplementary material for: Heparin-based hydrogel scaffolding alters the transcriptomic profile and increases the chemoresistance of MDA-MB-231 triple-negative breast cancer cells
Source: Biomater Sci. 2020 Feb 13;8(10):2786–96. doi: 10.1039/c9bm01481k (PMC7497406; doi:10.1039/c9bm01481k)
Supplement: Supplementary file 2 [file BM-008-C9BM01481K-s002.zip › Supplementary File 4/HP-B hydrogel + EGF (s)vControl/Pathways/my_analysis.Gsea.1545201170987/HALLMARK_P53_PATHWAY.html]

Details for gene set HALLMARK\_P53\_PATHWAY[GSEA]

|  || Dataset | expr.class.cls#HYDEGF\_versus\_CONTROL.class.cls#HYDEGF\_versus\_CONTROL\_repos |
| Phenotype | class.cls#HYDEGF\_versus\_CONTROL\_repos |
| Upregulated in class | CONTROL |
| GeneSet | HALLMARK\_P53\_PATHWAY |
| Enrichment Score (ES) | -0.2780078 |
| Normalized Enrichment Score (NES) | -1.3247736 |
| Nominal p-value | 0.027848102 |
| FDR q-value | 0.06641673 |
| FWER p-Value | 0.642 |
Table: GSEA Results Summary

  

Fig 1: Enrichment plot: HALLMARK\_P53\_PATHWAY      
 Profile of the Running ES Score & Positions of GeneSet Members on the Rank Ordered List

  

| PROBE | DESCRIPTION (from dataset) | GENE SYMBOL | GENE\_TITLE | RANK IN GENE LIST | RANK METRIC SCORE | RUNNING ES | CORE ENRICHMENT || 1 | PVT1 | na |  |  | 16 | 3.655 | 0.0219 | No |
| 2 | DEF6 | na |  |  | 112 | 2.865 | 0.0348 | No |
| 3 | DDB2 | na |  |  | 266 | 2.430 | 0.0419 | No |
| 4 | SLC3A2 | na |  |  | 461 | 2.166 | 0.0451 | No |
| 5 | ATF3 | na |  |  | 473 | 2.154 | 0.0580 | No |
| 6 | IRAK1 | na |  |  | 503 | 2.115 | 0.0696 | No |
| 7 | FBXW7 | na |  |  | 672 | 1.926 | 0.0728 | No |
| 8 | TNFSF9 | na |  |  | 738 | 1.875 | 0.0811 | No |
| 9 | PDGFA | na |  |  | 813 | 1.811 | 0.0884 | No |
| 10 | UPP1 | na |  |  | 814 | 1.811 | 0.0997 | No |
| 11 | TRAF4 | na |  |  | 965 | 1.710 | 0.1025 | No |
| 12 | SESN1 | na |  |  | 967 | 1.709 | 0.1131 | No |
| 13 | PPP1R15A | na |  |  | 1051 | 1.653 | 0.1190 | No |
| 14 | WRAP73 | na |  |  | 1143 | 1.604 | 0.1242 | No |
| 15 | IER5 | na |  |  | 1276 | 1.523 | 0.1268 | No |
| 16 | BAIAP2 | na |  |  | 1394 | 1.470 | 0.1298 | No |
| 17 | ABCC5 | na |  |  | 1401 | 1.467 | 0.1386 | No |
| 18 | AEN | na |  |  | 1668 | 1.367 | 0.1331 | No |
| 19 | RAD9A | na |  |  | 1823 | 1.318 | 0.1332 | No |
| 20 | HRAS | na |  |  | 2033 | 1.257 | 0.1301 | No |
| 21 | TCHH | na |  |  | 2179 | 1.217 | 0.1300 | No |
| 22 | FOS | na |  |  | 2215 | 1.207 | 0.1357 | No |
| 23 | PLK2 | na |  |  | 2245 | 1.197 | 0.1416 | No |
| 24 | PLK3 | na |  |  | 2392 | 1.153 | 0.1411 | No |
| 25 | POM121 | na |  |  | 2676 | 1.086 | 0.1330 | No |
| 26 | DDIT3 | na |  |  | 2748 | 1.066 | 0.1359 | No |
| 27 | GADD45A | na |  |  | 2798 | 1.052 | 0.1399 | No |
| 28 | EPHA2 | na |  |  | 2860 | 1.038 | 0.1432 | No |
| 29 | DGKA | na |  |  | 2890 | 1.030 | 0.1481 | No |
| 30 | EPS8L2 | na |  |  | 2954 | 1.016 | 0.1511 | No |
| 31 | JAG2 | na |  |  | 3263 | 0.949 | 0.1408 | No |
| 32 | TSPYL2 | na |  |  | 3370 | 0.928 | 0.1410 | No |
| 33 | BAX | na |  |  | 3746 | 0.856 | 0.1266 | No |
| 34 | OSGIN1 | na |  |  | 3913 | 0.828 | 0.1230 | No |
| 35 | VDR | na |  |  | 4214 | 0.779 | 0.1121 | No |
| 36 | PTPN14 | na |  |  | 4266 | 0.771 | 0.1142 | No |
| 37 | RAB40C | na |  |  | 4279 | 0.770 | 0.1184 | No |
| 38 | NOTCH1 | na |  |  | 4284 | 0.769 | 0.1230 | No |
| 39 | FDXR | na |  |  | 4868 | 0.674 | 0.0965 | No |
| 40 | PLXNB2 | na |  |  | 4893 | 0.669 | 0.0994 | No |
| 41 | HEXIM1 | na |  |  | 5050 | 0.645 | 0.0953 | No |
| 42 | LIF | na |  |  | 5147 | 0.629 | 0.0941 | No |
| 43 | RXRA | na |  |  | 5268 | 0.611 | 0.0916 | No |
| 44 | TRIB3 | na |  |  | 5408 | 0.590 | 0.0880 | No |
| 45 | HSPA4L | na |  |  | 5521 | 0.573 | 0.0857 | No |
| 46 | IFI30 | na |  |  | 5554 | 0.570 | 0.0875 | No |
| 47 | JUN | na |  |  | 5642 | 0.555 | 0.0864 | No |
| 48 | HDAC3 | na |  |  | 5786 | 0.534 | 0.0822 | No |
| 49 | GPX2 | na |  |  | 5805 | 0.532 | 0.0846 | No |
| 50 | POLH | na |  |  | 5980 | 0.505 | 0.0786 | No |
| 51 | SPHK1 | na |  |  | 6182 | 0.478 | 0.0710 | No |
| 52 | PPM1D | na |  |  | 6219 | 0.474 | 0.0721 | No |
| 53 | KRT17 | na |  |  | 6332 | 0.459 | 0.0690 | No |
| 54 | NUPR1 | na |  |  | 6404 | 0.449 | 0.0681 | No |
| 55 | FOXO3 | na |  |  | 6580 | 0.427 | 0.0616 | No |
| 56 | NOL8 | na |  |  | 6770 | 0.399 | 0.0541 | No |
| 57 | CGRRF1 | na |  |  | 7024 | 0.370 | 0.0431 | No |
| 58 | CDK5R1 | na |  |  | 7158 | 0.350 | 0.0383 | No |
| 59 | ST14 | na |  |  | 7179 | 0.347 | 0.0394 | No |
| 60 | CYFIP2 | na |  |  | 7182 | 0.347 | 0.0415 | No |
| 61 | ADA | na |  |  | 7361 | 0.322 | 0.0341 | No |
| 62 | SLC35D1 | na |  |  | 7417 | 0.314 | 0.0332 | No |
| 63 | KLF4 | na |  |  | 7914 | 0.254 | 0.0087 | No |
| 64 | BLCAP | na |  |  | 8035 | 0.239 | 0.0039 | No |
| 65 | STEAP3 | na |  |  | 8475 | 0.192 | -0.0180 | No |
| 66 | INHBB | na |  |  | 8618 | 0.169 | -0.0244 | No |
| 67 | SLC7A11 | na |  |  | 8638 | 0.165 | -0.0244 | No |
| 68 | IP6K2 | na |  |  | 8710 | 0.162 | -0.0271 | No |
| 69 | CCND3 | na |  |  | 8864 | 0.140 | -0.0343 | No |
| 70 | XPC | na |  |  | 8906 | 0.135 | -0.0356 | No |
| 71 | RALGDS | na |  |  | 8952 | 0.126 | -0.0372 | No |
| 72 | RRP8 | na |  |  | 8953 | 0.126 | -0.0364 | No |
| 73 | SFN | na |  |  | 9071 | 0.119 | -0.0418 | No |
| 74 | NUDT15 | na |  |  | 9073 | 0.118 | -0.0411 | No |
| 75 | PRKAB1 | na |  |  | 9337 | 0.084 | -0.0545 | No |
| 76 | ANKRA2 | na |  |  | 9598 | 0.062 | -0.0677 | No |
| 77 | AK1 | na |  |  | 9668 | 0.052 | -0.0710 | No |
| 78 | MAPKAPK3 | na |  |  | 9673 | 0.052 | -0.0709 | No |
| 79 | CDKN2AIP | na |  |  | 9712 | 0.048 | -0.0726 | No |
| 80 | SLC19A2 | na |  |  | 10072 | 0.009 | -0.0914 | No |
| 81 | HBEGF | na |  |  | 10340 | -0.028 | -0.1053 | No |
| 82 | TPD52L1 | na |  |  | 10368 | -0.032 | -0.1065 | No |
| 83 | HMOX1 | na |  |  | 10412 | -0.037 | -0.1086 | No |
| 84 | WWP1 | na |  |  | 10566 | -0.046 | -0.1163 | No |
| 85 | CSRNP2 | na |  |  | 10614 | -0.053 | -0.1185 | No |
| 86 | PHLDA3 | na |  |  | 10695 | -0.064 | -0.1223 | No |
| 87 | PMM1 | na |  |  | 10773 | -0.076 | -0.1258 | No |
| 88 | EI24 | na |  |  | 11069 | -0.116 | -0.1406 | No |
| 89 | ITGB4 | na |  |  | 11077 | -0.118 | -0.1403 | No |
| 90 | STOM | na |  |  | 11213 | -0.124 | -0.1466 | No |
| 91 | DNTTIP2 | na |  |  | 11215 | -0.125 | -0.1459 | No |
| 92 | RGS16 | na |  |  | 11341 | -0.139 | -0.1516 | No |
| 93 | ERCC5 | na |  |  | 11786 | -0.201 | -0.1737 | No |
| 94 | PRMT2 | na |  |  | 11822 | -0.208 | -0.1742 | No |
| 95 | CD81 | na |  |  | 11825 | -0.208 | -0.1730 | No |
| 96 | MKNK2 | na |  |  | 12029 | -0.222 | -0.1823 | No |
| 97 | RNF19B | na |  |  | 12235 | -0.251 | -0.1915 | No |
| 98 | PROCR | na |  |  | 12401 | -0.272 | -0.1985 | No |
| 99 | CASP1 | na |  |  | 12497 | -0.286 | -0.2017 | No |
| 100 | KIF13B | na |  |  | 12501 | -0.287 | -0.2001 | No |
| 101 | MDM2 | na |  |  | 12590 | -0.296 | -0.2029 | No |
| 102 | RETSAT | na |  |  | 12706 | -0.315 | -0.2070 | No |
| 103 | BAK1 | na |  |  | 12757 | -0.324 | -0.2076 | No |
| 104 | CCNK | na |  |  | 12768 | -0.326 | -0.2061 | No |
| 105 | DCXR | na |  |  | 12972 | -0.336 | -0.2146 | No |
| 106 | RAP2B | na |  |  | 13225 | -0.374 | -0.2256 | No |
| 107 | CCP110 | na |  |  | 13280 | -0.382 | -0.2260 | No |
| 108 | SDC1 | na |  |  | 13322 | -0.390 | -0.2257 | No |
| 109 | TRIAP1 | na |  |  | 13427 | -0.407 | -0.2287 | No |
| 110 | NINJ1 | na |  |  | 13435 | -0.408 | -0.2265 | No |
| 111 | MXD1 | na |  |  | 13508 | -0.414 | -0.2277 | No |
| 112 | ISCU | na |  |  | 13541 | -0.419 | -0.2268 | No |
| 113 | RHBDF2 | na |  |  | 13619 | -0.428 | -0.2282 | No |
| 114 | RRAD | na |  |  | 13718 | -0.444 | -0.2306 | No |
| 115 | MXD4 | na |  |  | 13783 | -0.455 | -0.2311 | No |
| 116 | FAS | na |  |  | 13787 | -0.456 | -0.2284 | No |
| 117 | TAX1BP3 | na |  |  | 13833 | -0.463 | -0.2279 | No |
| 118 | TCN2 | na |  |  | 13902 | -0.472 | -0.2285 | No |
| 119 | SEC61A1 | na |  |  | 13905 | -0.472 | -0.2257 | No |
| 120 | BTG2 | na |  |  | 14115 | -0.493 | -0.2336 | No |
| 121 | RAD51C | na |  |  | 14225 | -0.509 | -0.2362 | No |
| 122 | TGFB1 | na |  |  | 14260 | -0.515 | -0.2348 | No |
| 123 | ZMAT3 | na |  |  | 14465 | -0.551 | -0.2421 | No |
| 124 | CCND2 | na |  |  | 14645 | -0.580 | -0.2479 | No |
| 125 | PTPRE | na |  |  | 14865 | -0.614 | -0.2555 | No |
| 126 | CDKN1A | na |  |  | 14882 | -0.616 | -0.2526 | No |
| 127 | PCNA | na |  |  | 14963 | -0.629 | -0.2528 | No |
| 128 | SP1 | na |  |  | 15137 | -0.660 | -0.2578 | No |
| 129 | RB1 | na |  |  | 15219 | -0.672 | -0.2579 | No |
| 130 | LDHB | na |  |  | 15225 | -0.674 | -0.2540 | No |
| 131 | EPHX1 | na |  |  | 15301 | -0.687 | -0.2536 | No |
| 132 | APAF1 | na |  |  | 15368 | -0.700 | -0.2527 | No |
| 133 | RCHY1 | na |  |  | 15440 | -0.709 | -0.2521 | No |
| 134 | H2AFJ | na |  |  | 15502 | -0.722 | -0.2508 | No |
| 135 | CTSD | na |  |  | 15631 | -0.749 | -0.2528 | No |
| 136 | ACVR1B | na |  |  | 16066 | -0.837 | -0.2704 | No |
| 137 | DDIT4 | na |  |  | 16203 | -0.871 | -0.2722 | No |
| 138 | IL1A | na |  |  | 16315 | -0.902 | -0.2724 | Yes |
| 139 | TPRKB | na |  |  | 16375 | -0.918 | -0.2698 | Yes |
| 140 | ZFP36L1 | na |  |  | 16467 | -0.939 | -0.2687 | Yes |
| 141 | GM2A | na |  |  | 16579 | -0.964 | -0.2685 | Yes |
| 142 | HINT1 | na |  |  | 16616 | -0.975 | -0.2644 | Yes |
| 143 | RPL36 | na |  |  | 16730 | -1.006 | -0.2640 | Yes |
| 144 | RPL18 | na |  |  | 16788 | -1.023 | -0.2607 | Yes |
| 145 | F2R | na |  |  | 16819 | -1.032 | -0.2558 | Yes |
| 146 | TSC22D1 | na |  |  | 16950 | -1.072 | -0.2560 | Yes |
| 147 | TRAFD1 | na |  |  | 16979 | -1.082 | -0.2507 | Yes |
| 148 | TAP1 | na |  |  | 16995 | -1.089 | -0.2447 | Yes |
| 149 | TP63 | na |  |  | 17056 | -1.103 | -0.2410 | Yes |
| 150 | ABHD4 | na |  |  | 17099 | -1.112 | -0.2363 | Yes |
| 151 | TP53 | na |  |  | 17262 | -1.165 | -0.2375 | Yes |
| 152 | FUCA1 | na |  |  | 17274 | -1.170 | -0.2308 | Yes |
| 153 | TM7SF3 | na |  |  | 17290 | -1.178 | -0.2243 | Yes |
| 154 | TGFA | na |  |  | 17302 | -1.182 | -0.2175 | Yes |
| 155 | PITPNC1 | na |  |  | 17399 | -1.213 | -0.2150 | Yes |
| 156 | HIST1H1C | na |  |  | 17421 | -1.223 | -0.2085 | Yes |
| 157 | SOCS1 | na |  |  | 17552 | -1.279 | -0.2074 | Yes |
| 158 | DRAM1 | na |  |  | 17740 | -1.352 | -0.2088 | Yes |
| 159 | BTG1 | na |  |  | 17748 | -1.358 | -0.2007 | Yes |
| 160 | CTSF | na |  |  | 17752 | -1.360 | -0.1924 | Yes |
| 161 | CD82 | na |  |  | 17789 | -1.376 | -0.1857 | Yes |
| 162 | CCNG1 | na |  |  | 17820 | -1.396 | -0.1786 | Yes |
| 163 | SERTAD3 | na |  |  | 18045 | -1.509 | -0.1809 | Yes |
| 164 | TOB1 | na |  |  | 18307 | -1.689 | -0.1841 | Yes |
| 165 | RPS12 | na |  |  | 18376 | -1.733 | -0.1769 | Yes |
| 166 | BMP2 | na |  |  | 18629 | -1.962 | -0.1780 | Yes |
| 167 | S100A10 | na |  |  | 18646 | -1.987 | -0.1664 | Yes |
| 168 | APP | na |  |  | 18704 | -2.052 | -0.1566 | Yes |
| 169 | FAM162A | na |  |  | 18708 | -2.062 | -0.1439 | Yes |
| 170 | ABAT | na |  |  | 18799 | -2.192 | -0.1350 | Yes |
| 171 | S100A4 | na |  |  | 18812 | -2.210 | -0.1219 | Yes |
| 172 | NDRG1 | na |  |  | 18853 | -2.277 | -0.1098 | Yes |
| 173 | PERP | na |  |  | 18938 | -2.423 | -0.0991 | Yes |
| 174 | TM4SF1 | na |  |  | 18955 | -2.472 | -0.0846 | Yes |
| 175 | TXNIP | na |  |  | 18972 | -2.510 | -0.0698 | Yes |
| 176 | RPS27L | na |  |  | 19061 | -2.776 | -0.0571 | Yes |
| 177 | SAT1 | na |  |  | 19134 | -3.131 | -0.0414 | Yes |
| 178 | VAMP8 | na |  |  | 19170 | -3.509 | -0.0214 | Yes |
| 179 | VWA5A | na |  |  | 19175 | -3.665 | 0.0012 | Yes |
Table: GSEA details [plain text format]

  

Fig 2: HALLMARK\_P53\_PATHWAY      
 Blue-Pink O' Gram in the Space of the Analyzed GeneSet

  

Fig 3: HALLMARK\_P53\_PATHWAY: Random ES distribution      
 Gene set null distribution of ES for **HALLMARK\_P53\_PATHWAY**
